# Developing a Hybrid Platform for Emergency Remote Education of Nursing Students in the Context of COVID-19

**DOI:** 10.3390/ijerph182412908

**Published:** 2021-12-07

**Authors:** Hiromi Kawasaki, Satoko Yamasaki, Md Moshiur Rahman

**Affiliations:** Division of Nursing Science, Graduate School of Biomedical and Health Sciences, Hiroshima University, Hiroshima 734-8553, Japan; morisato@hiroshima-u.ac.jp (S.Y.); moshiur@hiroshima-u.ac.jp (M.M.R.)

**Keywords:** nursing students, COVID-19, remote learning, hybrid learning

## Abstract

Due to the COVID-19 pandemic, many nursing students are being taught remotely. Remote learning has drawbacks, such as decreased motivation for learning and difficulties conveying the instructor’s intentions. Strategies that compensate for the shortcomings of remote learning should be identified. This study aimed to evaluate the understanding of the knowledge use and awareness of negotiation methods through cases and teaching tools in nursing student classes on environmental assessment and daily life support, and to examine whether supplementary assistance can compensate for the drawbacks of remote learning. This study used a mixed-method design, and included 59 second-year nursing students attending an environmental assessment course in 2021. Students’ knowledge use and awareness of negotiation methods were evaluated using self-assessment worksheets before and after the class. The pre- and post-class scores were compared using the Wilcoxon signed-rank test. The mean knowledge score increased significantly during the study period (*p* < 0.001). Students acquired awareness of how to use the knowledge gained during class and negotiation awareness by observing role play, factors that strengthen motivation when learning alone. This study provides insight into the potential of class supplements to compensate for the deficits of remote learning. Supplementing the shortcomings of remote learning should be a priority and may be a focal point of hybrid learning.

## 1. Introduction

The COVID-19 pandemic has greatly affected learning. Since the academic year of 2020, university classes in Japan have been held remotely [1,2]. Students who started university in 2020 were unable to visit the campuses of their schools and took nearly all of their classes remotely for an entire year. As a result, many university students experienced anxiety [3,4], which had not subsided a year later [5]. Nursing students also participated in remote learning, which had unprecedented repercussions [5]. 

The merits and drawbacks of remote education have been extensively debated [6,7]. Its merits [8] include learning at one’s own pace, efficiency, and ability to save time by not commuting. Meanwhile, the drawbacks of remote learning [9] include an insufficient discussion that impedes motivation maintenance, its dependence on each student’s capacity for self-regulation, an unstable system for confirmation of the learned content through testing, and difficulties in accurately communicating the instructor’s intentions [10]. Moving forward, there is a need to take full advantage of the merits of remote learning while simultaneously compensating for its drawbacks [11]. 

The ability to observe other people and infer their feelings is honed through face-to-face encounters. Face-to-face encounters also facilitate relationship building based on coordination, manners, and trust. Remote learning and teleworking will decrease the opportunities to learn skills, such as noticing things through observing others, observing one’s work surroundings, learning from what others are doing [12], and learning the job by following others. It is important for people to learn how to understand others’ emotional states from the time of their childhood [13,14]. Moreover, insight into the emotional state of others is an important skill that nursing students should acquire. Negotiating has vital importance for the work process [15] and requires the ability to determine the circumstances that others are in, which is a skill of critical significance for nurses. 

In the time of the COVID-19 pandemic, college students were enrolled when the internet was already widespread. They are different from those who have started their life with the internet as new technology. They are characterized by a tendency to not speak directly with others but rather to communicate using email [16,17]. The insufficient amount of face-to-face learning has led to a situation in which students’ generation has even fewer chances to read others’ emotions [18]. Notably, COVID-19 has provided researchers with an opportunity to re-evaluate the significance of schools, including the development of human relations [19]. Nursing instructors should consider the students’ characteristics when structuring the curricula of nursing students. Identifying the behavioral characteristics of the students’ generation, which places great importance on efficiency, will also facilitate their acceptance of training. Moreover, nursing instructors need to provide clear instructions to students from this generation [16]. 

Two of the drawbacks of remote learning—the fact that the instructor’s ideas are not accurately communicated and the insufficient amount of discussion—interfere with one of the main tasks in nursing: advocacy. Conversations with patients and allied health professionals are not as successful when only the main points are communicated [20]. Nurses need to be skilled in observing how their superiors behave and to learn the skills they observe. Public health nurses, in particular, are in a position where they participate in policy creation for the health issues faced by local residents; thus, they need political skills to advocate for local residents [21].

Remote learning is associated with learning by oneself and the need for motivation to learn alone. Solving problems of insufficient motivation and self-regulation that result from taking classes by oneself is dependent on the skills of an individual [22]. As there is insufficient testing to determine the mastery of the material and inadequate practical skills, it is difficult to determine whether students have learned the material. The plan-do-check-act (PDCA) cycle has been unconsciously mastered by students over the course of their education and experience. According to the PDCA cycle, students observe the circumstances surrounding the main points and devise ways to deal with various circumstances [10]. However, learning the PDCA cycle can be inefficient in remote environments [10]. In face-to-face lessons, it is necessary to incorporate them actively. 

Students themselves also have a sense of anxiety about the future [3,4]. There is a need to provide supplementary support for class cohorts experiencing a large amount of remote learning to relieve students’ anxiety [23]. In the future, there may be an increasing amount of hybrid learning. Solving the problems that arise during continuous remote learning should be one of the focal points of hybrid learning [24]. 

In the current study, attempts to resolve the issue were conducted face-to-face in a period when COVID-19 cases were predicted to decrease. Assessment of classes, including supplementary student assistance of remote learning due to COVID-19, allowed instructors to seek potential solutions to the problems presented by remote learning in addition to assessing the achievement goal of regular classes. We evaluated regular lessons by Bloom’s three domains’ goals [25]. We have set goals that invent for the shortcomings of remote learning in addition to regular lessons goals.

This study aimed to evaluate the students’ understanding of how to use knowledge and communication methods by using mixed methods through cases and teaching tools in classes on environmental assessment and daily life support to nursing students and to examine the potential of supplementary assistance compensating for the drawbacks of remote learning.

## 2. Materials and Methods

### 2.1. Study Design

This study used longitudinal mixed methods, including a quasi-experimental design that compared before and after a single-group class. Qualitative data were analyzed through the means of content analysis.

### 2.2. Recruitment

The participants were nursing students who attended an environmental assessment course, a teaching unit of classes in basic nursing skills. Basic nursing skills classes are taught during June of the second year in the curriculum of the School of Nursing of Hiroshima University. The analyzed class took place in 2021.

### 2.3. Intervention in the Nursing Students

**Figure 1:**
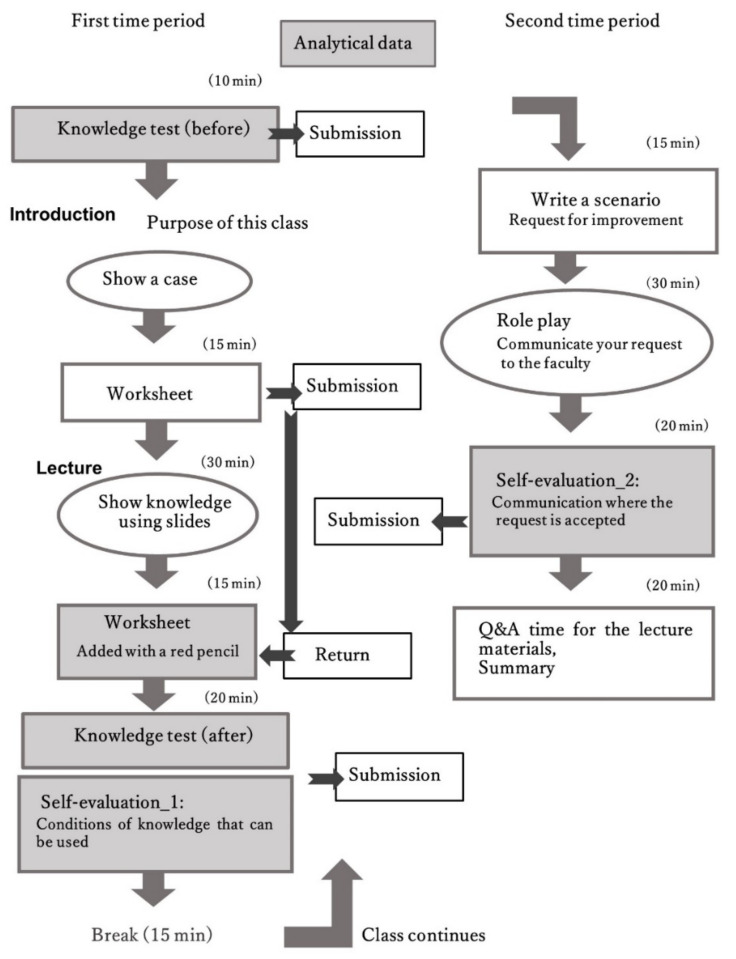
Teaching tools and order of their use.

The intervention took place in one class in the nurses’ second year of 2021. It was a regular 180-min continuous face-to-face lesson. The duration of the first period and the second period were 90 min each. They were connected by a 15-min break (Figure 1). Content has been added to the regular lesson tasks to make up for the shortcomings of distance learning.

### 2.4. Overview of the Class 

Originally, this class focused on the environmental assessment of people’s daily lives and hospital rooms as the main environmental assessment skills, and potential problems have been investigated to prevent health obstacles [10]. During the COVID-19 pandemic, the goals of the class did not change. In 2021, short-term face-to-face instruction was permitted after the emergency, and remote learning was introduced due to the COVID-19 pandemic. Since remote learning may continue in the future, the class included supplements to compensate for learning deficits caused by the remote learning format, and it was designed to allow a smooth transition to future skill learning. Specifically, the way knowledge could be used was considered to allow students to maintain their motivation even when learning alone. Moreover, the class was designed to facilitate students’ understanding of communication styles used when making requests of others and to enable a better understanding of how to determine the circumstances others are in (Figure 1). 

#### 2.4.1. Class Goal

Whereas during 2020 the students were educated remotely, the students could be educated face-to-face in 2021. In this 2021 class, the following specific goal was set according to Bloom’s three domains: “Comprehend the effects of an environment on health and understand preventative methods”. The knowledge required to achieve this goal was defined as the core competencies [26] in the basic nursing education course. The following items were selected from the core competencies and used as the class goal and knowledge base: health, knowledge of mental and physical mechanisms and operations, ability to assess individuals in the context of their daily lives, knowledge of basic human needs and self-care, and ability to utilize the environment in which people live when making assessments [10]. The central goal of the class was to understand healthcare in evacuation shelters during disasters. This was subdivided into specific goals following Bloom’s three domains as follows: (1) understanding the environmental conditions necessary for normal life (cognitive); (2) developing an interest in assessments involving an individual’s environment (affective); and (3) ability to suggest ways to prevent health issues (psychomotor) [25]. 

The compensational goals for the insufficient elements of remote learning included the following: (1) cognitive, understanding how to use knowledge (motivation to learn even when one is alone); (2) affective, having an interest in mastering knowledge (motivation to learn even when one is alone); and (3) psychomotor, understanding the features of communication in accordance with goals.

The goals and required knowledge were evaluated using 11 items selected from the core competencies included in the Japanese primary nursing curriculum [26] and Sphere standards [27] (Table 1).

#### 2.4.2. Knowledge Retention Rate 

Based on the “flipped classroom” method, a test was conducted at the start of the class using 11 items selected from the core competencies [26] and Sphere standards [27] in the basic nursing education course learned during the students’ first year of training (Table 1). As shown in Figure 1, a case is described, and the worksheet is used by students to produce estimates of what would happen at evacuation shelters and to complete the basic and improvement contents. After receiving the class, students corrected their worksheets by themselves. When this task was completed, students took the same knowledge test once again. Then, after completing the “how to use knowledge” section of the worksheet, the students performed a self-evaluation (Self-evaluation_1). 

#### 2.4.3. Communication to Achieve a Goal

The next task, added specifically in 2021, is to consider the conditions required to realize the worksheet-listed points needing improvement by requesting the help of key persons (played by a teacher). First, a scenario for making requests was created, and the students engaged in roleplaying. If a request convinced the key persons, they replied, “Yes, I will do it.” Five students were selected by the teacher to roleplay in front of all the students. The students considered the features of the requests that received a response of “Yes, I will do it” and recorded the results (Self-evaluation_2). 

#### 2.4.4. Details of the Case 

One day in June, while students were attending a lecture, an evacuation order was issued by Hiroshima City at 15:00, because heavy rains had caused the river near the university to rise above dangerous levels (bankfull stage). Residents had already evacuated to the university’s gymnasiums. The students evacuated to the gymnasium of a junior high school neighboring the university, where each person was given a bottle of mineral water (500 mL) and a blanket. Windows could not be opened because of heavy rains, and lights went out due to power outage. The shelter temperature was 28 °C, with a humidity of 90%.

### 2.5. Analytical Methods

All submitted materials were assigned an ID linked to the students who submitted them. Knowledge test scores were used for quantitative analysis. Scores on the knowledge test were determined based on one-point per-correct-answer for a maximum score of 11. The scores were compared using a two-sided Wilcoxon signed-rank test. Statistical analyses were performed using IBM SPSS Statistics (version 25.0; IBM Corp, Armonk, NY, USA) with a significance level of 0.05.

Student descriptions were qualitatively compared before and after the lecture by score change. Additionally, the contents of the worksheets of the students with the greatest change in their scores were examined quantitatively and qualitatively. Self-evaluation_1 and Self-evaluation_2 were analyzed using a qualitative descriptive content analysis. The analyses were performed individually and matched by three researchers to confirm their validity.

### 2.6. Ethical Considerations

The purpose, methodology, and data handling procedures of this study were explained to the students. They were informed that participation was voluntary, and they retained the right to refuse participation or to withdraw from the study. The participants provided written informed consent, and only the data of the consenting students were analyzed. All data were anonymized by assigning identification numbers unrelated to the corresponding participant. This study was approved by the Ethical Committee for Epidemiology of the Hiroshima University (accession no. E-1778-2) and funded by the Grants-in-Aid for Scientific Research Program (KAKENHI), Japan (grant number 17H04467).

## 3. Results

**Figure 2:**
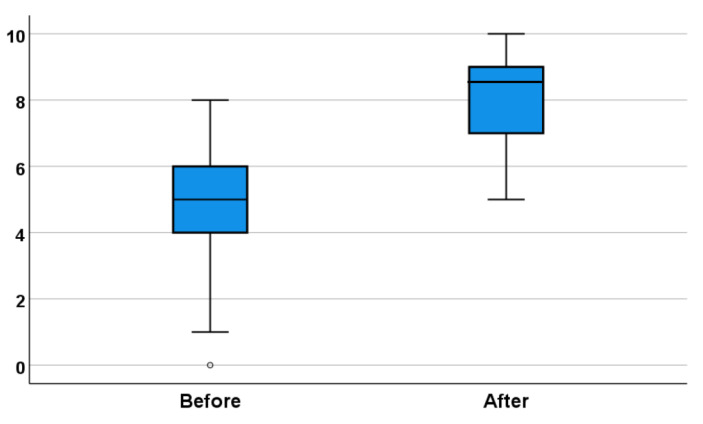
Knowledge test score distribution before and after courses.

In this study, 59 students provided consent to participate. The mean knowledge scores were 4.69 prior to the course and 8.25 (*p* < 0.001) after the course (Figure 2). 

The largest difference between the pre- and post-class scores was 10 (one person, Student 1), whereas the second largest difference was 6 (four students, Students 2–5). Table 2 shows the analysis results of the students’ descriptions with the largest difference in knowledge scores before and after the lecture as an example. Student 1 added a priority to the description in the worksheet. Specific numerical values and countermeasures have been added (Table 2). The increase in knowledge throughout the course led to more concrete and detailed descriptions and considerations. By the other students (Student 2–5), the statement “Students who can walk should use the toilets elsewhere” was replaced with “Priority will be given to increasing the number of women’s toilets.” Specific figures, reasons and improved plans were listed. Noise, privacy, distance from others, and light items were added. Specific numerical values were shown for the baseline and plans for improvement.

Next, we extracted 104 phrases from the students’ statements in the “How to use knowledge” Self-evaluation_1 section of the worksheet to determine how knowledge was used. The statements were divided into seven categories with the highest number of phrases (46) in the category titled “Knowledge is used for countermeasures.” The students wrote that knowledge was used for the management of humidity, distance from others, and the environment. The following categories were next: “Knowledge indicates an objective situation” (16 phrases); “Knowledge is used to compare with the status quo” (12 phrases); “Knowledge overlaps with real life” (11 phrases); “Knowledge is used for judgment” (6 phrases); “Knowledge is not just numbers” (6 phrases); and “Knowledge is used as a basis” (3 phrases) (Table 3). In the category “Knowledge is used for countermeasures”, many students compared their knowledge and current circumstances. The category “Knowledge indicates an objective situation” is required in situational explanations, such as when requesting the cooperation of others (Table 3). As knowledge increased throughout the course, the motivation to learn and acquire knowledge alone also increased as a result of reflection. After role-playing scenarios, in which they requested improvements based on the results of environmental assessments, the students focused on the method used to make the requests in cases in which the request was accepted.

Students wrote in Self-evaluation_2 “Why the request was accepted”. Six categories were extracted from 81 phrases in Self-evaluation_2: presentation of clear grounds (25), easy-to-execute proposal (19), specific proposal (15), sharing the situation with the other party (10), communication from a standpoint (9), and presentation of absolute necessity (3) (Table 4). The circumstances should be accurately communicated and compared to some standard (knowledge) so that the other party could perceive the need for improvement. In the presence of a clear grounds category, it was stated that a polite request linked to the grounds is easier to accept, and that specific numbers and standards were used to convey the reason for the request.

## 4. Discussion

Cases and learning tools for environmental assessment regular classes have been modified to make up for the shortcomings of remote learning. The supplementary content was the understanding of using knowledge and communication methods according to the purpose. These shortcomings were commonplace in face-to-face classes, which were added as attempts to fabricate these in remote learning as a motivation to learn by a person. However, the association between the characteristics of students [24] and remote learning developed a problem manifestation in COVID-19. We verified the achievements from the student’s description and confirmed that there was a possibility of supplementation. We found that knowledge was significantly increased, insight into students’ perceptive ability was gained, and students’ negotiation awareness was enhanced by observing role play. A previous study reported that the goals of traditional classes could be achieved sufficiently in remote learning [10,11]. Here, we demonstrated that the goal for the supplementation of remote learning has been achieved. Knowledge increased significantly by the end of the class. Students completed the worksheet to help them realize that knowledge and improved content were involved, and students’ statements were abstracted. Based on the additional writings provided by students who showed large score differences, we found that students had added contents presented in the lectures. The learning methods and values were recorded. Methods of utilization for knowledge included the use of knowledge to make judgments, to make improvements, and to request cooperation. Methods of learning included the use of knowledge repeatedly on a daily basis. Student additions clearly show the use of knowledge. Values were rumor rejection, observation, and communication. The statement that knowledge rejects rumors is necessary for observation and is of particular value when used for communication. The correct knowledge is one of the ways to protect people from infodemics [28]. It can be sufficient to motivate students to acquire knowledge.

Some of the drawbacks of remote learning are the lack of opportunity to see and learn from others, and to reinforce the technical acquisition of communication. In this study, the students learned face-to-face for a short time. They confirmed and abstracted that cooperation was required, and that requests should be communicated. Students used a limited number of roles to learn communication styles. In addition, they found that not only clear knowledge is needed for negotiation, but that polite words suitable for negotiation should also be used. Advocacy is to address the concerns of residents and speak for satisfying their demands [29]. Student awareness is worth playing this role. Moreover, understanding the other party, also referred to as “sharing the situation with the other party,” is needed. Notably, sharing circumstances, communicating politely, and wording can only be done when communicating face-to-face [30]. It is important to communicate circumstances accurately, compare knowledge, and ensure that the other party is aware of the request for improvement. Requests also require knowledge and communication [31].

In the basic nursing skills units, in environmental assessments conducted as part of environmental adjustments, it was possible to add goals and achieve the additional goals to compensate for the insufficiencies of remote learning. The important factors are cognitive (understanding the ways to use knowledge and motivation for learning alone), affective (interest in mastering knowledge and motivation for learning alone), and psychomotor (taking advantage of the characteristics of communication used when making requests). In order to achieve this goal, it is essential for teachers to provide clear instructions to students’ characteristics. We implemented it in basic nursing skills units. In other classes, we acknowledge that it is possible to compensate for the shortcomings of remote learning by adding the following elements: a consideration of the characteristics of communication according to the situation in which knowledge was used.

Previous studies have also identified both successes and challenges associated with the remote teaching of nurses during the COVID-19 pandemic [32,33]. Moreover, they emphasized the need for additional support for nursing students during this challenging time [34]. There was evidence that technical support in remote learning for students was also provided [23]. Furthermore, our findings are consistent with other studies that have identified the positive effects of hybrid learning in the context of the COVID-19 pandemic [35]. We must use remote lessons and face-to-face lessons appropriately, depending on the content and context of teaching [36].

Our study has several limitations. This study included a cross-sectional design and a relatively small sample size, which was also based on a single class. It is necessary to verify the degree of effectiveness of motivational cognition of knowledge usage, the degree of motivation to learn alone, and effective deadlines. Further studies are needed to address these aspects.

## 5. Conclusions

The current study findings show that, in nursing students, supplementary assistance with remote learning could compensate for its drawbacks. Specifically, supplementary assistance could lead to specific awareness of students on how to use the knowledge gained during the class, and acquisition of negotiation skills by observing role-play. In other words, it is possible to make up for the shortage of remote learning by incorporating a sort of ingenuity into regular lessons.

## Figures and Tables

**Table 1:** Knowledge test questions.

Category	Question ID (Shorthand)	Question [Unit]
Reference values: Interpersonal environment	Q1 (interpersonal distance)	How much distance [m] should be maintained in interpersonal interactions, whether in public or social settings?
	Q2 (illuminance)	How bright should patients’ rooms be in a hospital ward? Please give respective values for daytime and nighttime in lux [lx].
	Q3 (noise limits)	What are the reference levels for community noise in the daytime and nighttime? Please give each value in decibels [dB].
	Q4 (temperature)	What temperature should be maintained indoors during summer to feel comfortable? [°C]
	Q5 (humidity)	What humidity should be maintained indoors during summer to feel comfortable? [%]
Reference values: Physiological/internal	Q6 (discomfort index)	Which of the above values is perceived as uncomfortable?
	Q7 (stressful colors)	Which colors provoke stress?
	Q8 (water intake)	How much water does a person need per day [mL/day]?
	Q9 (urinations)	How many times does a person urinate per day?
	Q10 (caloric requirements)	How many calories does a person need per day, that is, what is their estimated energy requirement? Write the value [kcal/day] for a young adult male or female in their 20s of (physical) activity level II.
	Q11 (sleep time)	How many hours should a person sleep to stay healthy [h/day]?

**Table 2:** Greatest score differences between the beginning and end of the class.

Score Increase Until the End	ID	Possible Health Problems (Numbers Indicate Student Priorities)		Reason		Improvement Plan	
		Before the Lecture	After the Lecture	Before the Lecture	After the Lecture	Before the Lecture	After the Lecture
Score increase is 10.	Student 1	There is not enough space to sleep.	Priority ②	Currently, the size for one person is less than 1 tatami mat.	Two tatami mats are required per person.	Open an empty classroom	Currently, half of the people cannot be accommodated.
		There are not enough toilets.	Priority ③	Exceeding capacity	Insufficient women’s toilets	Students who can walk should use the toilets elsewhere.	Priority will be given to increasing the number of women’s toilets.
		Humidity becomes high.	Priority ④ Room temperature also rises.	Because it exceeds the capacity, the humidity will be quite high due to the rainy season.		Open the windows and ventilate	Install a cold air conditioner or a blower. People are crowded in one place.
		There is not enough light at night.		Power outage		Use the light of your smartphone instead.	Place a reflector where it is easy to trip.
			Priority ① Run out of water		Everyone needs 7.5–15 L of water daily; thus, if the evacuation period is extended, the water reserved in advance will be insufficient.		Water saving Request to institutions such as prefectures and cities

**Table 3:** Content extracted from Self-evaluation_1.

Code	Subcategory	Category
	Consider the amount of payment from the required amount (14)	Knowledge is used for countermeasures (46)
Understand the surrounding situation and determine the required amount (5)		
Prevent confusion by human behavioral characteristics (knowledge)		
Analyze the current problems and formulate concrete improvement measures		
(Knowledge) is used to think of the best solution (7)		
Assess and improve the environment		
	Used to manage interpersonal distance (7)	
Stress can be reduced by freeing up the necessary space (3)		
Capacity is determined by interpersonal distance (knowledge) (4)		
	Used to judge the amount of water (7)	
The amount of water supplied is determined by the amount of water required (5)		
Think about actions based on the amount of water required		
Request a shortage from the amount of water in a day		
	Judge the status of excretion (5)	
Toilet is managed based on past cases (2)		
Think about installing a toilet based on knowledge (2)		
The place to excrete is important (knowledge)		
Take measures to reach an appropriate temperature (3)	Used for temperature and humidity control (4)	
Reduce the humidity from which mold and germs grow		
Loud voice creates noise (2)	Noise management (3)	
Maintain noise standards		
Knowledge prioritizes and facilitates action	Set priorities (2)	
Use knowledge to prioritize the implementation of improvements		
Judge the current situation by the appropriate brightness and bring it closer to the actual situation	Ensure the required brightness (2)	
Set brightness to standards		
Knowledge is used to remove discomfort (countermeasure)	Eliminate the inconvenience of evacuees (2)	
Eliminate the inconvenience caused to evacuees using knowledge		
Compare with actual temperature/humidity values (2)	Knowledge is used for assessment (7)	Knowledge indicates an objective situation (16)
Assess and improve the environment (3)		
Judge that the amount of water consumed is low		
Compare knowledge with the current situation and act to satisfy physiological needs		
Use your knowledge to justify and improve	Knowledge is used when seeking cooperation (6)	
Request as a concrete numerical value based on solid knowledge, not your own sensory demand (4)		
When collecting supplies (knowledge required)		
When setting up a bed (knowledge required)	Knowledge is used as a basis (3)	
(According to knowledge) Noise is quite annoying		
(According to knowledge) The amount of water required is unexpectedly large (it turns out)		
Judge the current situation and bring it closer by the appropriate interpersonal distance (4)	Knowledge is compared with the current situation and used for shelter management (8)	Knowledge is used to compare with the status quo (12)
Ensuring the required amount by physiological standards		
Compare environmental standards with current status (2)		
The problem can be judged by the amount of water to be secured		
Compare knowledge with current status for disease prevention	Knowledge is compared with the current situation and used for health management (4)	
Assess and improve the environment		
Judgment and improvement of the current situation based on knowledge (2)		
For knowledge to be used, it actually has a connecting image (3) Reference value (knowledge) reminds us of a task	Have a real image (to make knowledge available) (5)	Knowledge overlaps with real life (11)
Used to imagine the environment		
Knowledge needs to be reviewed for quick judgment	Review (to make knowledge available) (3)	
To use knowledge, learn iteratively		
About 80% of knowledge can be used		
Knowledge may actually be different	Use knowledge in daily life (to make it available) (3)	
To use knowledge, use it in your life		
To use knowledge, think and use		
Use knowledge to compare and improve the desired environment	Use knowledge to think about ways to improve (3)	Knowledge is used for judgment (6)
Use for improvement		
Analyze the current problems and formulate concrete improvement measures		
Plan how to keep the rising temperature and humidity appropriate	Use knowledge to judge problems (3)	
Clarify problems from the standard and improve according to the standard (2)		
Combine knowledge and utilize it comprehensively	Understand the rationale as well as the numbers (4)	Knowledge is not just numbers (6)
Get knowledge along with evidence		
Specific numbers are needed for knowledge		
Get knowledge along with rationale		
You need the ability to see objectively	Observation and communicative competence are required (to use knowledge) (2)	
Communication skills are also required		
Knowledge is the basis	Knowledge is power (3)	Knowledge is used as a basis (3)
(Knowledge) corrects unfounded remarks		
Knowledge reinforces ideas		

**Table 4:** Content extracted from Self-evaluation_2.

Why Was the Improvement Request Not Accepted (Accepted)? (Phrase Extraction)	Subcategory	Category
Because I clearly communicated the rationale and the reason why I wanted you to do so (6)	*Clearly convey the rationale*	Presentation of clear grounds (25)
A polite request linked to the grounds is easier to accept	*Clearly justify*	
I told you exactly what the benefits of improvement would be	*Explain the benefits*	
Communicate (necessity) properly	*Communicate the need*	
I was speaking with grounds of whether it was a problem (15)	*Clearly state the rationale*	
It is easy to be recognized when there is something that seems to be feasible (5)	*Show a viable plan*	Easy-to-execute proposal (19)
Can be improved by on-the-spot judgment that does not require new substances	*Show a plan that can be implemented in the current situation*	
It was accepted because it was easy to practice (12)	*Show a plan that is easy to realize*	
It is easy to recognize improvement requests that can be implemented immediately by individual actions	*Show a plan that is easy to judge*	
The points to be improved were clear and easy to implement (11) It was specifically proposed	*Show a concrete plan*	Specific proposal (15)
Specific suggestions for making or improving your requirements	*Speak specifically*	
He stated concrete improvement measures and compromises (3)	*Show multiple plans*	
Explain the rationale for determining that you need to improve now	*There is a basis to judge*	Sharing the situation with the other party (10)
I was told that the rationale was clear and that it was really necessary	*The need is communicated*	
Give an explanation that can share the need (6)	*Share the need*	
It is necessary to explain in a quantity and number that makes it easy to grasp a concrete image The priority of the requesting evacuee and the priority of the city staff were the same	*Explain by number/quantity* *Priority matches*	
Choose a word	*Choose a word*	Communication from a standpoint (9)
Polite language when making a request (5)	*How to speak*	
Polite and rude tone (2) Can enthusiasm be transmitted to the other party	*Use polite words* *Communicate enthusiasm*	
It was the content and grounds that people would receive as the minimum required	*Explain the high need*	Presentation of absolute necessity (3)
Very important for maintaining human life (2)	*Communicate the high need*	

## Data Availability

The data presented in this study are available from the corresponding author upon reasonable request. The data are not publicly available because of the need to maintain the participants’ anonymity and data confidentiality.
